# Antidiabetic Effects of Tea

**DOI:** 10.3390/molecules22050849

**Published:** 2017-05-20

**Authors:** Qiu-Yue Fu, Qing-Sheng Li, Xiao-Ming Lin, Ru-Ying Qiao, Rui Yang, Xu-Min Li, Zhan-Bo Dong, Li-Ping Xiang, Xin-Qiang Zheng, Jian-Liang Lu, Cong-Bo Yuan, Jian-Hui Ye, Yue-Rong Liang

**Affiliations:** 1 Tea Research Institute, Zhejiang University, Hangzhou 310058, China; 21516103@zju.edu.cn (Q.-Y.F.); qsli@zju.edu.cn (Q.-S.L.); 21516097@zju.edu.cn (X.-M.L.); ryqiao@zju.edu.cn (R.-Y.Q.); kexiaonao@163.com (R.Y.); 21616096@zju.edu.cn (X.-M.L.); xqzheng@zju.edu.cn (X.-Q.Z.); jllu@zju.edu.cn (J.-L.L.); 2 Wenzhou Vocational College of Science and Technology, Wenzhou 325035, China; dozhanbo@gmail.com; 3 National Tea and Tea product Quality Supervision and Inspection Center (Guizhou), Zunyi 563100, China; gzzyzj_2009@vip.sina.com; 4 Yuyuanchun Tea Limited, Jufeng Town, Rizhao 276812, China; yuyuanchun@126.com

**Keywords:** *Camellia sinensis*, tea catechins, tea polysaccharides, caffeine, diabetes mellitus, epidemiological analysis

## Abstract

Diabetes mellitus (DM) is a chronic endocrine disease resulted from insulin secretory defect or insulin resistance and it is a leading cause of death around the world. The care of DM patients consumes a huge budget due to the high frequency of consultations and long hospitalizations, making DM a serious threat to both human health and global economies. Tea contains abundant polyphenols and caffeine which showed antidiabetic activity, so the development of antidiabetic medications from tea and its extracts is increasingly receiving attention. However, the results claiming an association between tea consumption and reduced DM risk are inconsistent. The advances in the epidemiologic evidence and the underlying antidiabetic mechanisms of tea are reviewed in this paper. The inconsistent results and the possible causes behind them are also discussed.

## 1. Introduction

Diabetes mellitus (DM) is a threat to human health and it was the 6th leading cause of death in the world in 2015 [1] and the 7^th^ leading cause of death in the USA in 2011 [2]. The number of people with DM was estimated to be 382 million in 2013, and it was predicted that by 2035 around 600 million people could develop DM [3], causing disabilities, economic hardship and even death. There are two types of DM. Type 1 DM (T1DM) is a metabolic disease characterized by an insulin secretory defect due to the autoimmune destruction of the beta cells in the pancreas [4] and type 2 DM (T2DM) is a long term metabolic disorder characterized by high blood sugar (hyperglycemia), insulin resistance, and relative lack of insulin [5]. T2DM is the most common form and it accounts for 90–95% of all DM cases [6,7,8]. A national survey conducted in 2010 showed that the weighted prevalence of T2DM among Chinese adults aged 18 years and above was 9.7% [9]. Three out of four people with DM live in low- and middle-income countries and they can’t afford necessary drugs such as insulin and/or other medicaments [10]. As both a serious threat to human health and global economies DM is at crisis levels and one of the biggest public health challenges in the 21st century.

Tea is a common beverage consumed daily in many parts of the world. It is classified into unfermented tea (green tea, white tea), semi-fermented tea (oolong tea) and fully fermented tea (black tea and pu’erh tea). The predominant chemical components in unfermented tea are catechins and caffeine, while in semi-fermented and fully fermented tea teatheaflavins, thearubigins and caffeine predominate. Catechins, caffeine and theaflavins have been confirmed to possess a broad range of biological activities [11,12]. Tea has been suggested to decrease blood glucose levels and to protect pancreatic β cells in diabetic mice. As a result the development of antidiabetic medications from tea and its extracts is increasingly receiving attention. However, human epidemiological studies linking tea consumption and DM risk have shown inconsistent results. In this review, we examine the possible role of tea consumption in modulating the risk of DM, as well as the possible mechanisms behind the observed associations and inconsistencies.

## 2. Epidemiologic Evidences

A series of population-based cohort studies have showed that tea consumption was associated with reduced risk of DM. A survey involving 8821 adults (51.4% female) conducted in Krakow (Poland) showed that higher tea consumption were negatively associated with metabolic syndrome, with an odds ratio (OR) of 0.79 (95% confidence intervals (CI) 0.67–0.92), after adjusting for potential confounding factors. Among specific components of metabolic syndrome, tea consumption was negatively associated with central obesity and fasting plasma glucose [13]. A 10-year follow-up study in The Netherlands involving 918 incident T2DM cases which were documented from among 40,011 participants showed that tea consumption was inversely associated with the hazard of T2DM, with an OR of 0.63 (95% CI: 0.47–0.86) for >5.0 cups of tea per day (*p* = 0.002). Total daily consumption of at least three cups of tea reduced the risk of T2DM by approximately 42% [14]. An 11.7-year follow-up survey in the UK involving 5823 British people (4055 men and 1768 women) showed that there was an association between tea intake and reduced risk of T2DM, with a hazard ratio (HR) of 0·66 (95% CI: 0.61–1.22; *p* < 0.05) after adjustment for age, gender, ethnicity and social status [15]. A 5-year follow-up study involving 17,413 Japanese people (6727 men and 10,686 women) from 40 to 65 years of age showed that consumption of green tea was inversely associated with risk of T2DM after adjustment for age, sex, body mass index, and other risk factors. Multivariable OR for DM among participants who frequently drank six cups of green tea per day were 0.67 (95% Cl: 0.47–0.94), compared with those who drank less than one cup per week [16]. A study involving 71,239 Danish women revealed that moderate first trimester tea intake was not associated with increased risk of gestational DM and may possibly have a protective effect [17]. A cross-sectional study involving 452 T2DM participants conducted in a community-based specialized care center in Pakistan showed that prevalence of uncontrolled DM was approximately 39% and higher consumption of tea was independently associated with uncontrolled DM, with an OR: 1.5 (95% CI: 1.0–2.2) [18].

Many meta-analysis studies have revealed the effects of tea consumption on the reduced risk of DM. A study including 608 T2DM patients carried in China showed that tea drinking could alleviate the decrease of fasting blood insulin (1.30 U/L, 95% CI: 0.36–2.24) and reduced waist circumference in more than 8-week intervention [19]. Data from 18 studies including 457,922 participants showed that high intakes of decaffeinated tea were significantly associated with reduced risk of incident diabetes [20]. Daily tea consumption (≥3 cups/day) is associated with a lower T2DM risk. However, further studies are needed to enrich related evidence, especially with regard to types of tea or sex [21].

Various kinds of tea showed different effects on DM. Consumption of unfermented green tea or semi-fermented oolong tea was considered to protect against the development of T2DM in Chinese men and women. Green tea consumption was associated with a lower risk of impaired fasting glucose (IFG), while oolong tea consumption was associated with a lower risk of impaired glucose tolerance (IGT). A U-shaped association was observed, subjects who consumed 16–30 cups of green and oolong tea per week had the lowest ORs for IFG and IGT, respectively [22], suggesting that green tea and oolong tea showed antidiabetic effects through different mechanisms. The chemical composition of teas varies with the degree of fermentation. The major bioactive component in unfermented green tea is epigallocatechin gallate (EGCG), but in the fully fermented black tea and the semi-fermented oolong tea the major active ingredients are the theaflavins and thearubigins [23,24]. IFG and IGT are two different states in insulin secretion and insulin resistance [25]. IFG involves severe hepatic insulin resistance with near-normal or normal muscle insulin resistance, while IGT has marked muscle insulin resistance with mild hepatic insulin resistance. Both IFG and IGT are characterized by a decrease in early-phase insulin secretion, while subjects with IGT also have an impaired late-phase insulin secretion. Both conditions contribute to the development of T2DM [26]. Green tea consumption was particularly associated with a lower risk of IFG, probably as a result of its high level EGCG, which had insulin mimetic effects. First, EGCG inhibits the hepatic glucose production, and promotes tyrosine phosphorylation of the insulin receptor and insulin receptor substrate-1 (IRS-1). Second, EGCG controls gluconeogenesis by suppressing the expression of genes phosphoenolpyruvate carboxykinase (*PEPCK*) and glucose-6-phosphatase (*G6Pase*), ameliorates cytokine-induced β-cell damage and improves insulin sensitivity [27]. Third, EGCG regulates the expression of genes involved in the insulin signal transduction pathways and glucose uptake [28]. Oolong tea in particular lowered the risk of IGT, in which theaflavins may be the contributor. First, theaflavins inhibit α-glucosidase activity, resulting in decrease in glucose production at the intestine [29]. Second, compared to green tea, oolong tea contains higher levels of caffeine which increases glucose transporter IV expression [30]. An ecological study using a systematic data-mining approach to investigate the potential statistical relationship between consumption of fully fermented black tea and epidemiological indicators around the world showed that high black tea consumption was significantly correlated to low DM prevalence [31]. A single dose of black tea decreased peripheral vascular resistance across upper and lower limbs after a glucose load, accompanying by a lower insulin response (*p* < 0.05). Postprandial insulin response was attenuated by ~29% after tea intake (*p* < 0.0005) [32]. Chronic administration of the *Rauvolfia-Citrus* tea to overweight T2DM patients on oral anti-diabetic agents significantly improved the markers of glycaemic control and modified the fatty acid profile of skeletal muscle, without adverse effects or hypoglycaemia [33].

## 3. Protective Effects of Tea Against DM

### 3.1. Alleviation of Oxidative Stress

Oxidative stress is implicated in the pathogenesis of DM which is associated with distribution of cognitive functioning. Hyperglycemia-induced oxidative stress has been proposed as a cause of memory complications of DM including cognitive impairment. GTE showed antioxidant effects [34,35,36] and improved cognitive impairment in streptozotocin (STZ)-induced DM rats [37]. Daily intake of one cup (150 mL) to four cups (600 mL) of black tea improved oxidative stress biomarkers and decreased serum C-reactive protein level in DM patients [38].

Tea and tea extracts exhibit antioxidative effects through various pathways. First, antioxidants in tea can scavenge free radicals by directly acting on active oxygen species [35,39,40,41]. Second, tea components, especially the abundant tea polyphenols, chelate metal ions such as liver iron, preventing their participation in Fenton and Haber–Weiss reactions [41,42], a hydroxyl radical- forming process. Third, tea antioxidants increase the level of plasma antioxidants such as glutathione [36,38]. Fourth, tea bioactives suppress the activity of superoxidase by chelating plasma zinc and copper, the important cofactors of superoxidase [43]. Fifth, tea bioactives increase the biological activity of antioxidases including catalase (CAT), superoxide dismutase (SOD) [39], and glutathione peroxidase (GSH-Px) [36]. Sixth, tea catechins inhibit plasma protein carbonylation induced by hyperglycemia. Protein oxidative modifications are a major class of protein post-translational changes and contribute to cell dysfunction and human disease including T2DM. Carbonylation is an irreversible modification in oxidized proteins [36]. This explains why tea catechins showed beneficial effects against the redox impairment linked to hyperglycemic conditions.

There are many bioactive components in tea showing antioxidant activity, including catechins [23], gallic acid [44] and polysaccharides [45]. EGCG, the most abundant catechin compound, is a potent antioxidant in GTE [46]. Tea polysaccharides are another group of antioxidants in tea, and their antioxidant activities depend on monosaccharide composition and molecular weight [47]. The polysaccharides extracted from fully fermented black tea consisted of a higher proportion of low molecular weight fractions than those extracted from green tea and oolong tea, resulting in higher bioactivities [45]. Gallic acid concentration in tea increases with fermentation during tea processing, with 1.67 ± 0.06 mg/g in the unfermented green tea and 21.98 ± 1.03 mg/g in the fully fermented pu’erh tea [44].

### 3.2. Inhibition of α-Amylase and α-Glucosidase Activity

Controlling the sharp increase in postprandial blood glucose resulted from rapidly digesting and absorbing dietary carbohydrates is a challenge for DM patients. Dietary supplement of α-amylase and α-glucosidase inhibitors is an accepted clinical method for controlling the increase in postprandial blood glucose. Tea was confirmed to contain many natural products showing obvious inhibitory effects on α-glucosidase and α-amylase [48]. In vivo study on a starch ^13^C breath test on 28 healthy volunteers showed that GTE intake inhibited α-amylase activity, resulting in decreased absorption and digestion of starch [49]. The 50% inhibitory concentration (IC50) of green tea, black tea and oolong tea for inhibiting α-glucosidase was 2.82, 2.25 and 1.38 μg/mL equivalent polyphenols, respectively [50].

The bioactive components in tea such as catehcins, chlorogenic acid, caffeine and theaflavins show inhibitory effects on α-glucosidase and α-amylase activity, resulting in decreased plasma levels of glucose, lipid metabolites, and albuminuria [29,51,52], among which tea polyphenols showed major inhibitory potential [53,54,55]. Both α-amylase and a-glucosidase can be restrained in a non-competitive way. Structure-activity relationships of polyphenols by computational ligand docking showed that their inhibitory activity depends on the hydrogen bonds between the enzyme and hydroxyl groups in ring B or the condensate AC-ring, and the formation of a conjugated π-system that stabilizes the interaction with the active site [56]. A docking study showed green tea epicatechin gallate (ECG) presented stronger affinity than EGCG, with more number of amino acid residues involved in amylase binding with hydrogen bonds and Van der Waals forces [57].

The number of hydroxyl groups on the bioactive compounds is important for α-glucosidase inhibition. The structure of gallate group esterified at the 3-position of the C-ring of catechins is important and so the gallated catechins such as catechin gallate (CG), gaallocatechin gallate (GCG), ECG, and EGCG showed stronger inhibitory activity than their corresponding ungallated compounds catechin (C), gallocatechin (GC), epicatechin (EC) and epigallocatechin (EGC), respectively [55,58]. Black tea theaflavins constitute a group of pigments which are formed from the condensation of catechins during fermentation of black tea and they could strongly suppress the α-glucosidase activity. The α-glucosidase inhibitory effects of theaflavins decreased in the order of potency of theaflavin-3-*O*-gallate, theaflavin-3,3′-di-*O*-gallate, theaflavin-3′-*O*-gallate and theaflavin [29].

### 3.3. Improvement of Endothelial Disfunction

Endothelial dysfunction (ED) is considered to be the initial lesion of atherosclerosis which is the main etiology for mortality and a great percent of morbidity in DM patients. The ED is associated with the changes in the concentration of the chemical messengers produced by the endothelial cell and/or by blunting of the nitric oxide (NO)-dependent vasodilatory response to acetylcholine or hyperemia [59]. Factors associated with ED in DM patients include activation of protein kinase C, over expression of growth factors and/or cytokines, and oxidative stress. Green tea catechins (0.3–3.0 mg/mL) suppressed the decrease in nicotinamide adenine dinucleotide phosphate and NO in endothelial cells induced by high glucose (HG) [60], and so green tea EGCG acutely improved ED in patients with coronary artery disease [61].

The underlying mechanisms of endothelial improvement may be related to phosphatidyl inositol 3 kinase (PI3K)/protein kinase B (Akt) and mitogen-activated protein kinases (MAPK) signaling pathways. Catechin hydrate treatment prevents DM-induced vascular endothelial abnormalities through activation of PI3K signal and subsequent activation of endothelial nitric oxide synthase (eNOS) and generation of NO [62]. Tea EGCG induced endothelium-dependent vasodilation by a PI3K and Akt-dependent increase in eNOS activity [63]. Enhancing p38 MAPK could improve phosphorylation of the estrogen receptor α (ERα) on Ser-118, and stimulate eNOS activity. Polyphenol-induced eNOS activation required cotransfection with ERα subject to phosphorylation at Ser-118. Black tea polyphenols enhanced eNOS activity through p38 MAPK activation [64].

### 3.4. Modulation of Cytokines Expression

Cytokines are small-secreted proteins expressed mainly by immunocompetent cells, which contribute to inflammation as inflammatory mediators [65]. Inflammation induces and amplifies the immune assault against pancreatic β cells at early stages, then stabilizes and maintains insulitis at later stages. Inflammatory mediators suppress β cells function and subsequent apoptosis, even inhibit β cells regeneration and cause peripheral insulin resistance [66]. Β-Cell dysfunction and insulitis are key pathological events in DM, thus the modulation of cytokine expression is highly related to DM. Interleukin (IL), a tumor necrosis factor alpha (TNF-α), and interferon gamma (IFN-γ) are normally used in research as typical cytokines.

Tea and tea extracts may have protective effects against cytokine-induced injuries in β cells and insulin-producing cells. There was study showing that three cups (600 mL with 7.5 g) of black tea intake reduced pro-inflammatory cytokines as TNF-α in human, while the expressions of the anti-inflammatory cytokines such as IL-4, IL-5 and IL-10 were increased [67]. An in vitro study showed that black tea infusion (150 mL with 2.5 g) suppressed expression of the inflammatory cytokines TNF-α, IL-1β and IL-6, while it increased IFN-Υ, suggesting black tea had a selective pro-inflammatory cytokine that can also reduce IL-1β and IFN-Υ in insulinoma cell and inducible NO synthase (iNOS) gene expression suppressive effect [68]. EGCG, as an important component of tea catechins, inhibited the pro-inflammatory effects induced by cytokine that lead to a reduction of glucose-induced insulin secretion [69] through inhibiting NF-κB activation [27]. Furthermore, green tea can modulate cytokine expression in the periodontium and attenuate alveolar bone resorption in which the expression of pro-inflammatory cytokine TNF-α is reduced and the anti-inflammatory cytokine IL-10 is increased [70].

Pro-inflammatory cytokines can reduce glucose-induced insulin secretion and mitochondrial activity in cells. EGCG pretreatment of cells has an effect on mitochondrial electron transfer and energy production, and thus protects the insulin-producing cells through the mitochondrial pathway [69]. Recent studies have further indicated that green tea EGCG inhibits DNA methyltransferase activity by reactivating methylation-silenced genes and, consequently, IL-10 expression [71]. Most of the epidemiology and in vivo studies have showed that tea or tea extracts could reduce cytokines or have a selective effect. The concentration of EGCG is vital to its biofunction, as excessive concentration of EGCG may trigger other pathways leading to an invalid result.

### 3.5. Ameliorating Insulin Resistance

Insulin resistance, a key characteristic of T2DM, is characterized by impaired insulin-mediated glucose disposal in skeletal muscle, hepatic cells or adipose cells. Epidemiological studies have suggested that tea or tea extracts can decrease insulin resistance, with homeostasis model assessment of insulin resistance or insulin sensitivity, TG, glycemic, cholesterol, and lipid profiles as index [72,73,74]. DM decreases the acetylcholinesterase (AChE) activity on brain or erythrocytes membrane, which is a potential cause of insulin resistance. EGCG restored AChE activity to normal levels like insulin treatment [75].

The function of insulin is to induce PI3K sensitive phosphorylation of transcription factor FOXO1a that is sensitive to scavengers of free radicals. EGCG mimics insulin actions [76] and promotes nuclear efflux of FOXO1 in skeletal muscle, resulting in increase in insulin receptor (IR) sensitivity and decrease in insulin resistance [77]. Dysfunction of IR substrate, a family of docking molecules attaching to IR, may be the predominant molecular lesion signature of insulin resistance in liver [78]. EGCG can reduce Ser307 phosphorylation of IRS-1 to attenuate insulin signaling blockade through 5’-AMP-activated protein kinase (AMPK) activation pathway, eventually leading to reduction of insulin resistance [79,80]. Green tea GC, a kind of tea catechins, can stimulate Akt phosphorylation, instead of AMPK phosphorylation, to promote skeletal muscle glucose transport [81]. An in vivo study indicated that tea may ameliorate insulin resistance by increasing expression of glucose transporters IV [82], or alleviating oxidative stress by scavenging reactive oxygen species [83].

### 3.6. Antihyperglycemic Activity

As a hallmark of DM, chronic hyperglycemia could initiate impairment in diverse cell types [84] and were considered to be a main cause of diabetic complications like microvascular complications, neuropathy, retinopathy, stroke, nephropathy and peripheral vascular disease [85]. Intake of 1500 mL of the oolong tea (15 g brewed in 1500 mL boiling water) by T2DM patients for 1–2 weeks decreased the concentrations of plasma glucose and fructosamine [86]. The hypoglycemic effect of green tea is mainly due to its abundant polyphenols, especially catechins, which play a beneficial role in improving the glucose metabolism of DM, in which EGCG is the predominant antidiabetic active ingredient [87].

First, EGCG can regulate the expression of genes involving in insulin resistance. The sirtuin family of proteins is downregulated in cells with high insulin resistance, resulting in an increase in insulin sensitivity. A catechin-enriched GTE prevented glucose-induced survival reduction in *Caenorhabditis elegans* through activating adaptive responses mediated by gene SIR-2.1 [88], a gene encoding one of the sirtuin proteins.

Second, tea extracts regulate enzymes contributing to absorption of glucose. The α-glucosidase activity was inhibited by oral administration of black tea brew in both normoglycaemic and STZ-induced DM adult male Wistar rats, resulting in the impairment of glucose absorption from small intestine and the improvement of glucose tolerance [89]. Maltase was suppressed by oral administration of extract of LG tea (a co-fermented tea using green tea leaf and loquat leaf, 50 mg/kg) in maltose-loaded SD rats, showing a strong antihyperglycemic effect [90].

Third, EGCG stimulate glucose-induced insulin secretion. Dietary supplementation with EGCG at dosages of 2.5–10.0 g/kg of diet for 5–10 weeks in diabetic db/db mice or diabetic Zucker Diabetic Fatty rats increased blood insulin concentration in a dose-dependent manner [91], resulting in boost of oral glucose tolerance and reduction of plasma glucose levels. EGCG also improved pancreatic function and glucose-stimulated insulin secretion [91].

### 3.7. Improving Complications Associated with Hyperglycemia

DM is often accompanied by many hyperglycemia-associated complications, including dyslipidemia, cardiovascular diseases (CVD), albuminuria and nephropathy as well as liver damage. Bioactive components in tea can improve the diabetes complications.

Dyslipidemia is common in DM patients, which is associated with cardiovascular and atherosclerosis diseases and characterized by overweight and high levels of triglycerides (TG), total cholesterol (TC), low-density lipoprotein cholesterol (LDL-C), and low levels of high-density lipoprotein cholesterol (HDL-C) [92,93,94,95]. Animal tests and clinic studies showed that tea and its extract can alleviate the dyslipidemia. Tests on mice and SD rats revealed that dietary supplementation with green tea EGCG (1% feed weight) significantly reduced the weight gain and the adipose tissue weight caused by high fat diet. EGCG supplementation prevented diet-induced increases in body weight and in state plasma levels of glucose, TG, and leptin by decreasing fatty acid synthase (FAS) and acetyl-CoA carboxylase-1 mRNA levels in adipose tissue [96]. Furthermore, caffeine in tea might play a role in controlling body weight and dyslipidemia, because caffeine supplement (2.5–8.0% feed weight) could reduce the body weight and hepatosteatosis in mice overfed with zebrafish [97]. Test on T2DM patients showed that intake of chamomile tea (3 g/150 mL hot water) three times a day after meals for 8 weeks resulted in a considerable decrease in serum TG, TC, and LDL-C, compared to control group drinking water [98]. Tea consumption improves DM-induced CVD. The development of DM is a complex process, in which there is an intermediate state known as prediabetes characterized by a higher level of blood glucose than normal but below the level identified as DM [99]. Prediabetes is associated with CVD. White tea consumption ameliorated overall metabolic status of prediabetic rats and prevented most of heart-related deleterious effects by improving glucose tolerance and insulin sensitivity, increasing the level of cardiac lactate and acetate, decreasing the glucose transporters (GLUT1 and GLUT3) mRNA expression in cardiac tissue [100,101]. In addition to tea polyphenols, geraniol, a compound of tea aromatic volatiles, was confirmed to ameliorate impaired vascular reactivity in STZ-induced DM rats [102].

Tea and its extract ameliorate DM-associated albuminuria which is strongly associated with the increased risk of diabetic nephropathy. Oral administration of green tea or green tea poplyphenols reduced albuminuria in DM patients by decreasing podocyte apoptosis through activation of the WNT pathway [103]. Test on STZ-induced DM rats showed that that (+)-catechin and EGCG in green tea had renoprotective properties comparable with angiotensin-converting enzyme inhibitor (ACEi), which is the primary treatment option for patients with diabetic nephropathy [104].

GTE showed hepatoprotective properties in STZ-induced DM rats. Treatment with GTE (1.5%, *w/v*) improved histopathological and serum biomarkers in liver tissue in STZ-induced rats, including hepatic glucose, TG and TC levels as well as antioxidant activities [105].

### 3.8. Regulation of Gene Expression

Bioactive compounds in tea or tea extract play a role in controlling BG levels by regulating the expression of genes involving in glycometabolism and the insulin signaling (Irs) pathway. EGCG downregulated the genes involving in gluconeogenesis. Studies on H4IIE cells and db/db mice showed that EGCG downregulated the mRNA expression of gluconeogenic enzyme phosphoenolpyruvate carboxykinase, but upregulated the mRNA expression of glucokinase, an enzyme that facilitates phosphorylation of glucose to glucose-6-phosphate [91]. GTE at 1 g per kg diet increased the levels of glucose transporter family (Glut) genes Glut1, Glut4, Gsk3b, and Irs pathway gene Irs2 mRNA by 110, 160, 30, and 60% in the liver, respectively, and increased Irs1 by 80% in the muscle. GTE at 2 g per kg diet increased levels of Glut4, Gsk3b, and Pik3cb mRNA by 90, 30, and 30% in the liver and increased Glut2, Glut4, Shc1, and Sos1 by 80, 40, 60, and 50% in the muscle [28]. Dietary supplement of GTE or EGCG has the potential for beneficially modifying glucose and markedly enhancing glucose tolerance by regulating the expression of genes involving in the glucose uptake and Irs pathway.

EGCG or green tea regulate the expression of genes involving in the lipid metabolism and the pathways associated with ageing diseases. EGCG downregulated the genes involving in synthesis of fatty acids, triacylgycerol and cholesterol [91], resulting in reducing weight and alleviating DM complications. Advanced glycation end products (AGEs) are believed to play a causative role in the vascular complications of DM [106,107]. Nuclear factor erythroid-2-related-factor-2 (Nrf2) is a transcription factor that is encoded by the NFE2L2 gene in humans [108], which regulates the expression of antioxidant proteins which protect against oxidative damage triggered by inflammation and injury. Receptor activator of nuclear factor kappa-B ligand (RANKL) is a type II membrane protein and a member of the tumor necrosis factor (TNF) superfamily. RANKL can affect the immune system and control the bone regeneration and remodeling. RANKL is an apoptosis regulator gene which controls cell proliferation by modifying protein levels of Id4, Id2 and cyclin D1 [109]. Dietary supplement of EGCG attenuated the formation of AGEs in both plasma and liver, activated Nrf2, but inhibited the expression of receptor for AGE, compared to control mice without dietary supplement of EGCG [110]. Dietary green tea treatment decreased the expression of the osteoclastogenic mediator RANKL and pro-inflammatory cytokine TNF-α in STZ-induced DM rats, compared with the control group treated with water. Green tea also increased the expression of osteoprotegerin (a member of the TNF receptor superfamily), runt-related transcription factor 2 (RUNX-2, a key transcription factor associated with osteoblast differentiation) and anti-inflammatory cytokine IL-10 [70]. Dietary supplementation with green tea or EGCG could potentially contribute to nutritional strategies for the prevention of DM.

### 3.9. Alleviating Diabetes-Induced Damages of Neural Cells

Alzheimer's disease (AD) is a devastating neurodegenerative disorder. Increasing evidences implicate that DM is a risk factor for AD. Tea consumption has been associated with low prevalence of AD and severe cognitive impairment [111].

The brain is susceptible to glucose fluctuations and hyperglycemia-induced oxidative stress. Phytochemicals, such as polyphenols, l-theanine, polysaccharides and methylxanthines were considered to be responsible for the antidiabetic and neuroprotective properties of tea. Daily administration of tea in prediabetic Wistar rats improved the cerebral cortex neurons by inhibiting glucose metabolism in the cerebral cortex and reducing the cerebral cortex alanine content and the expression level of brain glucose transporters. Furthermore, regular consumption of tea has positive effects on DM-caused complications and restored the cerebral cortex antioxidant capacity and protein synthesis to normal levels in the cerebral cortex of prediabetic rats [112], contributing to an improvement of the cognitive function. GABA tea prevented the diabetic-induced cerebral cortex apoptosis by inhibiting the activity of FAS and by blocking the expression of mitochondrial apoptotic pathway components, such as FAS, pro-apoptotic t-Bid, Bax, cytosolic cytochrome c, and caspase-3, caspase-8 and caspase-9. GABA tea also reduced the diabetic-induced autophagy [113]. The formation of amyloid is associated with the development of diseases such as Huntington's chorea, Parkinson's disease and AD [114,115]. EGCG can effectively inhibit the formation of islet amyloid polypeptide (IAPP) and break down the amyloid formed by IPPA, which is closely related to the formation of T2DM [116].

GTE can modulate the analgesic effect of morphine in DM mice [117]. STZ can induce regurgitation, thermal hyperalgesia and abnormal pain [118,119,120] and DM decreased the analgesic effect of morphine [121]. However, co-administration of GTE (50 mg/kg·BW) and morphine (5 mg/kg·BW) enhanced the analgesic effect of morphine. A possible mechanism is considered to be the inhibitory effect of GTE on iNOS, resulting in a decrease in NO free radical levels [122]. Green tea EGCG also inhibited the expression of iNOS by suppressing the inhibitor of nuclear factor kappa B (NF-κB) and cytokines IL-1, resulting in activation of the NF-κB and inhibition of the IL-1β induced iNOS [123,124]. These experiments suggest that GTE enhanced the analgesic effect of morphine via its inhibitory effects on cytokines and iNOS. Furthermore, GTE alleviated the DM-induced vision disorder. The expression levels of glial fibrillary acidic protein, glutamine synthetase and oxidative retinal markers were increased in DM rats, resulting in vision disorder. However, green tea protected the retina against glutamate toxicity via an antioxidant mechanism [125].

### 3.10. Immunity Improvement and Anti-Inflammation

Inflammation is an important reaction associated with T2DM. GTE had suppressive effects on inflammatory transcription factors in STZ-induced DM rats. The expression of proinflammatory in retinae was significantly inhibited by oral administration of GTE (200 mg/kg·BW), as compared to control (*p* < 0.05) [40]. Dietary supplementation with EGCG at concentration 0.1% for 25 weeks in T2DM Goto-Kakizaki rats suppressed the expression of genes encoding proteins involved in inflammation such as IL-1β, TNF-α, IL-6, CD11s, CD18 and MCP-1 in adipose tissue [126]. Multiple low doses of STZ (MLD-STZ) induced diabetes via local and systemic pro-inflammatory cytokines [127]. GTE suppressed the expression of MLD-STZ-induced iNOS, in which EGCG was considered to play a protective role via blocking the cytokine-oxygen radical NF-κB (nuclear factor κB)-iNOS pathway [128]. It is considered that the anti-inflammatory activity of EGCG might be associated with decreased expression of inflammatory transcription factors including NF-κB, signal transducer and transcription activator, as well as suppressed pro-inflammatory factors such as IL-1β, TNF-α, iNOS, toll-like receptor 4, cyclooxygenase-2 and interferon γ [69,129,130,131,132,133].

## 4. Inconsistent Results

Although there were many in vitro studies showing that tea and tea extracts had antidiabetic effects, inconsistent results were also reported from in vivo and clinic studies. A double-blind, placebo controlled, randomized multiple-dose (0, 375, or 750 mg GTE or black tea extract per day for 3 months) study in adults with T2DM not taking insulin revealed that green tea or black tea extracts showed no hypoglycemic effect inT2DM adults [134]. A prospective cohort study including 4975 male workers over a median of 3.4 years of follow-up in Japan revealed that long-term consumption of oolong tea may be a predictive factor for new onset diabetes. Compared with those not consuming oolong tea, multivariable adjusted HR for developing diabetes were 1.00 (95% CI: 0.67–1.49) for those who drank one cup of oolong tea per day and 1.64 (95% CI: 1.11–2.40) for those drinking two or more cups per day. Fasting blood glucose increment per year was 0.11 mmol/L (95% CI: 0.09–0.12 mmol/L) for those who did not drink oolong tea, 0.12 mmol/L (95% CI: 0.09–0.15 mmol/L) for those who drank one cup per day and 0.15 mmol/L (95% CI: 0.11–0.18 mmol /L) for those who drank two cups per day, with a significant linear trend (*p* < 0.0001) [135]. A case-control study involved 600 newly diagnosed DM children and 536 randomly selected population-based children showed that the risk for T1DM was increased in the children who consumed one cup of tea (OR: 1.69, 95% CI: 1.21–2.37) or at least two cups daily (OR 2.59, 95% CI: 1.60–4.18) when adjusted for mother's education, child's age and child's sex [136].

DM is not a single diseases but a complex syndrome characterized by hyperglycemia resulting from altered carbohydrate, fat and protein metabolism. There were many factors leading to the inconsistent results on association of the tea consumption and the decreased DM risk.

First, dosage difference between animal test and clinic test resulted in great variation in hypoglycaemic effect between animal and clinic tests. When Xiaoke tea was consumed by STZ-induced DM mice at 20–50 times of the recommended clinical dose (per unit body weight), it produced a slowly generated antihyperglycaemic effect, without affecting insulin concentrations [137,138]. However, no significant effects on blood glucose and insulin concentrations were observed in T2DM patients who drank four cups (2.72 g tea bag infused in 250 mL freshly boiled water) of Xiaoke tea or green tea daily for four weeks [139].

Second, the antidiabetic effects of tea and tea extract were differentiated among the tested individuals. Tests on non-obese diabetic mice using water-soluble tea polysaccharide conjugates (TPC-W) and alkali-soluble tea polysaccharide conjugates (TPC-A) extracted from green tea showed that two out of 10 mice in non-obese diabetic groups treated with TPC-W or TPC-A exhibited diabetic symptoms compared with model control group, in which seven of 10 mice developed diabetes [140]. Analysis also showed a difference between men and women. High tea consumption was negatively associated with central obesity and fasting plasma glucose in women, but not in men [13]. The RR was 0.92 (95% CI: 0.84 to 1.00) for men, and 1.00 (95% CI: 0.96 to 1.05) for women. For Asians, the RR was 0.84 (95% CI: 0.71–1.00); but for Americans and Europeans, the RR was 1.00 (95% CI: 0.97–1.04). Tea consumption of more than three cups per day was associated with decreased T2DM risk in women, but not in men [21].

Third, changes in chemical composition of teas used in experiments lead to variation of test results. Partial removal of tea components might lead to changes in efficacy. There was test showing that total caffeine was associated with a reduced risk for T2DM [16]. Decaffeinated tea was prepared by partial removal of caffeine from tea leaf [141]. Although daily consumption of more than three cups of full tea could reduce risk of T2DM [14], intake of decaffeinated tea was not associated with the reduced T2DM risk [142]. The effect of non-fermented green tea on DM was differentiated from that of fully fermented black tea. It was shown that green tea consumption was associated with increased prevalence of DM (*p* = 0.0001), but black tea consumption showed no association with DM [143].

Fourth, many indicators can be used to assess antidiabetic effects of medicines, but individual medicine might play an antidiabetic effect through different pathways. PGC-1α, a key regulator of mitochondrial biogenesis and function, plays an important role in the improvement of insulin sensitivity by increasing fatty acids β-oxidation. Palmitate induced insulin resistance in C2C12 cells by decreasing PGC-1α protein expression. Rosiglitazone (RGZ), an anti-diabetic drug, could alleviate the palmitate-induced PGC-1α expression inhibition, while green tea EGCG had no significant effect on the expression of this gene. However, both RGZ and EGCG significantly improved glucose uptake in the C2C12 cells treated with palmitate, suggesting that RGZ and EGCG both exert their anti-diabetic activity by increasing insulin sensitivity, but with different molecular mechanisms. The effect of RGZ is mediated, at least partly, by increasing PGC-1α protein expression [144], while EGCG played its role through different pathway.

## 5. Conclusions and Future Expectations

Both in vitro and in vivo tests have confirmed that green tea catechins, black tea theaflavins and polysaccharides and caffeine in both green tea and black tea showed antidiabetic effects on T2DM. Most of the epidemiologic studies showed daily consumption of green tea, black tea and oolong tea and dietary supplements of EGCG have beneficial effects on T2DM. Table 1 summarizes the epidemiological evidence for the association between tea drinking and the risk of T2DM.

Tea and its extract play an antidiabetes role by alleviating oxidative stress, inhibiting α-amylase and α-glucosidase activity, improving endothelial disfunction, modulating cytokine expression, ameliorating insulin resistance, suppressing hyperglycemia, improving hyperglycemic complications, regulating signaling pathway involving in DM, enhancing immunity and alleviating diabetes-induced damages of neural cells.

The antidiabetic effect of tea depends on the bioactive compounds in tea. The chemical composition of teas varies with the tea cultivars and degree fermentation during tea processing, which lead to inconsistent antidiabetic results between various tests using teas from various sources. It is important to isolate purified individual bioactive compounds so as to test their antidiabetic effect individually. This will help to clarify the principal antidiabetic components in tea, and be interesting for improvement of tea processing.

Bioavailability is an important factor influencing the pharmaceutical effects of tea on diabetes [11]. The inconsistent antidiabetic effect of tea from tests on various individuals and populations might be related to the variation in bioavailability of tea components owing to differences in physiological status between tested individuals and populations. Encapsulating tea extract in chitosan nanoparticles was reported to be beneficial to stabilize tea bioactive components such as catechins in vivo, and to improve intestinal absorption [145]. Furthermore, co-administration of green tea EGCG with foods such as strawberry sorbet will reduce plasma EGCG [146]. Studies on in vivo adsorption mechanism of antidiabetic components in tea and development of methods to improve their bioavailability will be an important research topic in the future.

## Figures and Tables

**Table 1:** Epidemiological evidence for the association between tea drinking and the risk of T2DM.

Type of Study	Location	Tea Type	Number of Subjects	Main Results	References
Population based study	Krakow, Poland	Black tea	8821 adults (51.4% female)	Tea consumption was negatively associated with central obesity and fasting plasma glucose	[13]
Population based cohort study	Amsterdam, The Netherlands	Black tea green tea	10-year follow-up (40,011 participants)	Daily consumption of ≥3 cups of tea reduced the risk of T2DM by 42%	[14]
Prospective cohort study	London, UK	Black tea	11.7 years follow-up (4055 men and 1768 women)	Tea intake was associated with the reduced risk of T2DM, with a hazard ratio (HR): 0·66 (95% CI: 0.61–1.22; *p* < 0.05) after adjustment for age, gender, ethnicity and social status	[15]
Retrospective cohort study	Osaka, Japan	Green tea	5-year follow-up (6727 men and 10,686 women)	Drinking six cups of green tea per day was significantly associated with a lower risk for T2DM (OR = 0.67, 95% CI: 0.47–0.94)	[16]
Case-control study	Denmark	Black tea	Cases: 912, control: 70,327	Moderate first trimester tea intake were not associated with increased risk of gestational diabetes mellitus, but may have a protective effect	[17]
Community based study	Karachi, Pakistan	Black tea	452 T2DM participants	Prevalence of uncontrolled DM (UDM) was about 39% and higher consumption of tea was independently associated with UDM, with an OR: 1.5 (95% CI: 1.0–2.2)	[18]
Meta-analysis	China, South Korea, USA, Japan, Iran	Black tea, green tea, oolong tea	608 participants	Tea drinking could alleviate the decrease of fasting blood insulin (1.30 U/L, 95% CI: 0.36–2.24) and reduced waist circumference in more than 8-week intervention	[19]
Meta-analysis	USA, Japan, Singapore, Puerto Rico, UK, Finland	Black tea, green tea	457,922 participants	High intakes of decaffeinated tea were significantly associated with reduced risk of incident diabetes	[20]
Meta-analysis	12 countries including USA, Finland, Japan, UK, and etc	Oolong tea, green tea	761,949 participants	Daily tea consumption (≥3 cups/day) was associated with a lower T2DM risk	[21]
Cross-sectional study	Fujian, China	Oolong tea, green tea, black tea	4808 participants	Consumption of green or oolong tea may protect against the development of T2DM in Chinese men and women, particularly in those who drink 16–30 cups per week	[22]
Meta-analysis	A World Health Survey involving 50 countries	Black tea	More than 38,562 participants	High black tea consumption was significantly correlated to low DM prevalence	[31]
Cross-sectional study	Nijmegen, The Netherlands	Black tea	16 men	A single dose of black tea decreased peripheral vascular resistance (VR) across upper and lower limbs after a glucose load which was accompanied by a lower insulin response (*p* < 0.05). Postprandial insulin response was attenuated by ~29% after tea consumption (*p* < 0.0005)	[32]
Case control study	Denmark	Rauvolfia-Citrus tea	Cases: 11, control: 7	Chronic administration of the Rauvolfia-Citrus tea to overweight T2DM on OADs caused significant improvements in markers of glycaemic control and modifications to the fatty acid profile of skeletal muscle, without adverse effects or hypoglycaemia	[33]
